# Supplementary material for: Predicting the Area under the Plasma Concentration-Time Curve (AUC) for First Dose Vancomycin Using First-Order Pharmacokinetic Equations
Source: Antibiotics (Basel). 2023 Mar 23;12(4):630. doi: 10.3390/antibiotics12040630 (PMC10135334; doi:10.3390/antibiotics12040630)
Supplement: Supplementary file 1 [file antibiotics-12-00630-s001.zip › antibiotics-2264628-supplementary.pdf]

Supplementary Materials

Table S1: Data characteristics of adult and pediatric data sets

	**Adult data set**	**Pediatric data set**
N	10	14
Male (%)	80	64.3
Age (years)[a]	59.0 (45.8, 79.5)	6.4 (3.3, 10.7)
Weight (kg)[a]	61.0 (55.3, 67.3)	16.5 (12.8, 29.3)
Serum creatinine (mg/dL)[a]	1.27 (0.95, 1.60)	0.35 (0.28, 0.40)
Creatinine clearance[a]	43.5 (28.8, 92.5) mL/min	183.1 (148.2, 219.5) mL/min/1.73 m^2
Dose (mg/kg)[a]	29.9 (29.7, 30.0)	15.6 (14.7, 16.9)
Infusion period (minutes)	120	60
Sampling time during infusion (minutes after start infusion)	30, 60	30
Sampling time post infusion (minutes after the completed infusion)	0, 10, 20, 40, 60, 90, 120, and 240	0, 15, 30, 60, 120, 180, 240 and 300
Serum vancomycin measurement method	Fluorescence polarization immunoassay (AxSYM; Abbott Laboratories, Abbott Park, IL, USA)	*In vitro* chemiluminescent microparticle immunoassay, CMIA (ARCHITECT i-Vancomycin, Abbott Laboratories)

[a]Median (IQR)

Table S2: Agreement, bias and correlation results from adult data set using model 1

**Time points**		**Bland-Altman analysis**				**Correlation**		**Lin's coefficients**	
**C_1**	**C_2**	**Mean**	**Difference**		**95% limits of agreement**	**Pearson's r**	***P*-value**	**Rho_c**	***P*-value**
			**Mean (%)**	**SD**					
0	240	375.1	-104.0 (-27.7)	64.7	(-230.7, 22.7)	0.918	<0.001	0.575	<0.001
10	240	380.3	-93.8 (-24.7)	57.7	(-206.9, 19.3)	0.939	<0.001	0.643	<0.001
20	240	385.0	-84.4 (-21.9)	59.0	(-200.0, 31.3)	0.916	<0.001	0.686	<0.001
40	240	395.8	-62.6 (-15.8)	53.4	(-167.2, 42.0)	0.921	<0.001	0.802	<0.001
60	240	410.0	-34.3 (-8.4)	30.6	(-94.2, 25.7)	0.975	<0.001	0.937	<0.001
90	240	413.5	-27.2 (-6.6)	26.3	(-78.8, 24.3)	0.981	<0.001	0.958	<0.001
120	240	447.8	80.8 (18.0%)	124.6	(-163.4, 325.1)	0.871	0.002	0.679	<0.001

Table S3: Agreement, bias and correlation results from adult data set using model 2

Time points		Bland-Altman analysis				Correlation		Lin's coefficient	
C_1	C_2	Mean	Difference		95% limits of agreement	Pearson's r	*P*-value	Rho_c	*P*-value
			Mean (%)	SD					
0	240	417.5	-19.3 (-4.6)	60.2	(-137.3, 98.8)	0.899	<0.001	0.869	<0.001
10	240	412.9	-28.6 (-6.9)	54.0	(-134.4, 77.3)	0.928	<0.001	0.875	<0.001
20	240	414.7	-24.9 (-6.0)	55.3	(-133.3, 83.5)	0.918	<0.001	0.881	<0.001
40	240	422.2	-9.9 (-2.3)	50.0	(-107.9, 88.0)	0.929	<0.001	0.923	<0.001
60	240	433.6	13.0 (3.0)	30.0	(-45.0, 70.9)	0.976	<0.001	0.971	<0.001
90	240	436.1	18.0 (4.1)	30.0	(-40.8, 76.7)	0.976	<0.001	0.967	<0.001
120	240	468.2	121.7 (26.0)	121.0	(-115.5, 358.8)	0.878	0.002	0.607	<0.001

Table S4: Agreement, bias and correlation results from pediatric data set using model 1

Time points		Bland-Altman analysis				Correlation		Lin's coefficient	
C_1	C_2	Mean	Difference		95% limits of agreement	Pearson's r	*P*-value	Rho_c	*P*-value
			Mean (%)	SD					
0	240	111.9	-3.0 (-2.7)	13.4	(-29.1, 23.2)	0.974	<0.001	0.940	<0.001
15	240	107.1	-10.0 (-9.3)	13.8	(-36.7, 17.3)	0.967	<0.001	0.903	<0.001
30	240	105.0	-13.7 (-13.04)	11.3	(-35.8, 8.4)	0.974	<0.001	0.900	<0.001
60	240	103.9	-16.1 (-15.5)	10.8	(-37.2, 5.1)	0.971	<0.001	0.888	<0.001
120	240	103.3	-17.2 (-16.7)	10.5	(-37.8, 3.4)	0.969	<0.001	0.889	<0.001
180	240	105.0	-13.9 (-13.2)	15.5	(-44.3, 16.5)	0.932	<0.001	0.873	<0.001
0	300	115.3	3.9 (3.4)	10.2	(-16.2, 24.0)	0.977	<0.001	0.965	<0.001
15	300	109.3	-5.3 (-4.8)	9.9	(-24.7, 14.2)	0.98	<0.001	0.960	<0.001
30	300	106.7	-10.4 (-9.7)	7.5	(-25.2, 4.3)	0.986	<0.001	0.951	<0.001
60	300	105.1	-13.6 (-12.9)	7.5	(-28.3, 1.1)	0.985	<0.001	0.932	<0.001
120	300	103.3	-17.3 (-16.7)	9.6	(-36.0, 1.4)	0.977	<0.001	0.900	<0.001
180	300	99.6	-12.4 (-12.4)	11.9	(-35.7, 11.0)	0.964	<0.001	0.923	<0.001
240	300	108.0	-7.8 (-7.2)	22.7	(-52.3, 36.7)	0.958	<0.001	0.894	<0.001

Table S5: Agreement, bias and correlation results from pediatric data set using model 2

**Time points**		**Bland-Altman analysis**				**Correlation**		**Lin's coefficient**	
**C_1**	**C_2**	**Mean**	**Difference** Mean (%)	SD	**95% limits of agreement**	**Pearson's r**	***P*-value**	**Rho_c**	***P*-value**
0	240	125.1	23.4 (18.7)	14.7	(-5.5, 52.3)	0.949	<0.001	0.793	<0.001
15	240	117.5	11.3 (9.6)	14.5	(-17.1, 40.0)	0.954	<0.001	0.889	<0.001
30	240	114.1	4.3 (3.8)	11.3	(-17.8, 26.4)	0.969	<0.001	0.955	<0.001
60	240	111.8	-0.1 (-0.1)	11.4	(-22.6, 22.3)	0.964	<0.001	0.962	<0.001
120	240	109.6	-4.6 (-4.2)	11.6	(-27.4, 18.2)	0.963	<0.001	0.957	<0.001
180	240	113.3	2.8 (2.5)	20.5	(-37.4, 43.0)	0.883	<0.001	0.881	<0.001
0	300	128.1	29.6 (23.1)	13.2	(3.8, 55.3)	0.955	<0.001	0.759	<0.001
15	300	119.5	15.2 (12.7)	11.1	(-6.5, 36.9)	0.970	<0.001	0.894	<0.001
30	300	115.5	7.2 (6.2)	8.1	(-8.6, 23.0)	0.982	<0.001	0.966	<0.001
60	300	113.0	2.1 (1.9)	8.4	(-14.4, 18.7)	0.981	<0.001	0.979	<0.001
120	300	109.7	-4.4 (-4.01)	10.6	(-25.1, 16.2)	0.974	<0.001	0.966	<0.001
180	300	113.5	3.2 (2.8)	16.2	(-28.5, 34.9)	0.939	<0.001	0.933	<0.001
240	300	115.4	7.0 (6.1)	23.9	(-39.8, 53.8)	0.957	<0.001	0.890	<0.001

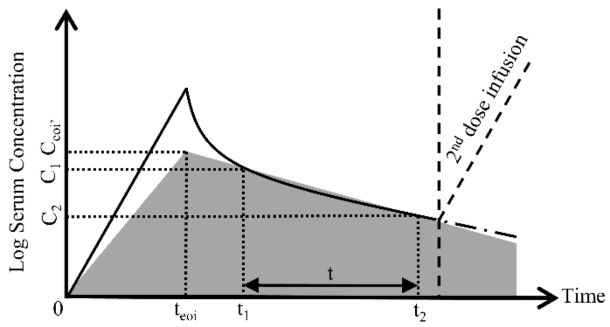

Figure S1: Expected area under the concentration curve calculated using model 1 compared to the expected first dose vancomycin concentration time profile

Abbreviations: C_1, first concentration measured at t_1; C_2, second concentration measured at t_2; $C_{eoi'}$, estimated end of infusion concentration; t, time difference between t_1 and t_2; t_{eoi}, end of infusion time

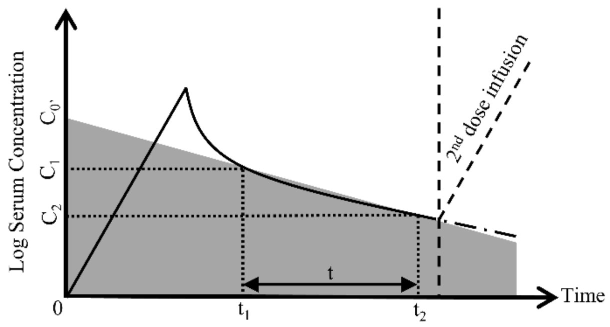

Figure S2: Expected area under the concentration curve calculated using model 2 compared to the expected first dose vancomycin concentration time profile

Abbreviations: $C_{0'}$, backward extrapolation peak concentration to the start of infusion; C_1, first concentration measured at t_1; C_2, second concentration measured at t_2; t, time difference between t_1 and t_2
